# Supplementary material for: Legume-nodulating rhizobia are widespread in soils and plants across the island of O‘ahu, Hawai‘i
Source: PLoS One. 2023 Sep 11;18(9):e0291250. doi: 10.1371/journal.pone.0291250 (PMC10495000; doi:10.1371/journal.pone.0291250)
Supplement: S1 Table — Samples at each site was obtained with permission from landowners. The GPS coordinates at these sites are not reported to protect the identity of each farm as per our sampling agreement. (DOCX) [file pone.0291250.s001.docx]

**S1 Table. Site location and characteristics of the soil samples in this study.**

| **Site Name** | **Latitude** | **Longitude** | **Land use** | **Soil Order** | **Number of samples** |
| --- | --- | --- | --- | --- | --- |
| Koko Crater Botanical Garden | 21.288450 | -157.681125 | semidisturbed | Andisols | 33 |
| University of Hawaii at Manoa campus | 21.297770 | -157.814775 | semidisturbed | Mollisols | 30 |
| Wa'ahila Ridge | 21.298363 | -157.809928 | undisturbed | Vertisols | 9 |
| Lyon Arboretum | 21.335955 | -157.804010 | semidisturbed | Andisols | 40 |
| Waimanalo Research Station | 21.336264 | -157.712403 | agricultural | Oxisols | 18 |
| West Oahu Farm |  |  | agricultural | Vertisols | 18 |
| North Oahu Farm 1 |  |  | agricultural | Oxisols | 12 |
| Poamoho Research Station | 21.545453 | -158.087915 | agricultural | Oxisols | 96 |
| North Oahu Farm 2 |  |  | agricultural | Oxisols | 100 |
| Waimea Ridge | 21.590490 | -157.954990 | undisturbed | Oxisols | 3 |
| Waimea Valley | 21.620300 | -158.014660 | undisturbed | Entisols | 3 |
| Waimea Botanical Garden | 21.633600 | -158.052090 | agricultural | Oxisols | 15 |

Samples at each site was obtained with permission from landowners. The GPS coordinates at these sites are not reported to protect the identity of each farm as per our sampling agreement.
